# Racial and Income Disparities in Health-Related Quality of Life among Smokers with a Quit Attempt in Louisiana

**DOI:** 10.3390/medicina55020048

**Published:** 2019-02-13

**Authors:** Yu-Hsiang Kao, Michael D. Celestin, Qingzhao Yu, Sarah Moody-Thomas, Krysten Jones-Winn, Tung-Sung Tseng

**Affiliations:** 1 Behavioral and Community Health Sciences, School of Public Health, Louisiana State University Health Sciences Center, New Orleans, LA 70112, USA; ykao1@lsuhsc.edu (Y.-H.K.); mceles@lsuhsc.edu (M.D.C.J.); SThoma@lsuhsc.edu (S.M.-T.); kjone8@lsuhsc.edu (K.J.-W.); 2 Biostatistics, School of Public Health, Louisiana State University Health Sciences Center, New Orleans, LA 70112, USA; qyu@lsuhsc.edu

**Keywords:** smoking, disparity, health-related quality of life, EQ-5D, quit attempt

## Abstract

*Background and objectives:* Smoking is associated with a lower health-related quality of life (HRQOL). However, there is little information about the association between HRQOL in relation to race, income, and smoking status. The present study aimed to assess the association between HRQOL and smoking status for those of different races and income levels. *Materials and Methods:* This study applied a cross-sectional design using data from the 2017 patient survey of the Louisiana Tobacco Control Initiative. We obtained 1108 responses from patients at eight Louisiana public hospitals. The EuroQol (EQ-5D) US index score assessed HRQOL. Smoking status was classified into four groups: never smoked, former smoker, current smoker with a quit attempt, and current smoker without a quit attempt. Multivariate linear regression analyses were used to estimate the HRQOL for black or African Americans and whites. *Results:* The patients were predominantly black or African American (58.9%) with lower-income (71.2%). Bivariate analyses showed that there were differences in income levels between black or African Americans and whites (*p =* 0.006). Moreover, black or African Americans (median = 0.80) had a higher mean of HRQOL than whites (median = 0.76). Among lower-income black or African Americans, current smokers with a quit attempt had a lower HRQOL than current smokers (coefficient = −0.12; *p <* 0.01). *Conclusions:* Racial and income disparities were evident with regards to HRQOL, with lower-income black or African Americans who were current smokers with a quit attempt having a lower HRQOL. Intervention programs for smoking cessation should target lower-income black or African American smokers who have a prior quit attempt and provide effective cessation services to help them quit smoking and improve their HRQOL.

## 1. Introduction

Smoking increases the risk of mortality and morbidity, often via cancer and other diseases of the lung and cardiovascular system [1,2,3,4,5]. Additionally, the annual economic cost of cigarette smoking in the US is estimated to be $300 billion [6], and health care spending attributable to smoking is estimated to be $1693 billion [7]. For current smokers of any age, there are many benefits from quitting smoking [2,4,8]. For example, quitting prolongs life expectancy [4] increases the quality of life [9,10,11,12] and reduces health care expenditures [13]. Therefore, encouraging current smokers to quit is an important public health issue.

There are disparities in not only cigarette smoking, [2] but also cessation behavior across groups defined by race, ethnicity, and socioeconomic status (SES) [14]. As determined by the Centers for Disease Control and Prevention, in 2015, 68.0% of adult smokers wanted to stop smoking, and 55.4% who had ever smoked made a quit attempt in the past year [14]. Furthermore, relative to whites, more African Americans reported interest in quitting (72.8% vs. 67.5%) and attempting to stop in the past year (63.4% vs. 53.3%) [14]. However, African Americans are less likely to successfully quit compared to whites (4.9% vs. 7.1%) [14]. Additionally, the high smoking rate (26.1%) for lower-income smokers could be a result of unsuccessful quit attempts, [14,15] because people with a higher SES have a higher rate of success in quitting smoking for at least one month [14,16]. Therefore, an issue for public health is to eliminate the disparity in smoking cessation for smokers across racial lines and SES.

The European Quality of Life—5 Dimensions (EQ-5D) questionnaire, developed by the EuroQol Group, measures health status in five dimensions, including mobility, self-care, usual activities, pain/discomfort, and anxiety/depression [17]. The EQ-5D, a subjective, patient-reported outcome instrument from the perspective of individuals, has been widely applied to measure health-related quality of life (HRQOL) [18]. HRQOL, an indicator of overall health, reflects an individual’s physical and mental health status that relates to their quality of life [18]. The EQ-5D has been validated [19] and has been used in clinical trials and health economic evaluations, such as health technology assessments, to assess HRQOL [20,21,22,23,24]. Moreover, it is widely known that health disparities exist in regards to racial minorities. Relative to whites, African Americans have worse general and disease-specific HRQOL [25,26]. However, the association between smoking status and preference-based scores on HRQOL measures is not well understood. Additionally, there are few investigations into the disparities between white and black or African American populations related to smoking status. Hence, it is worthwhile to assess HRQOL as it relates to smoking status, race, and income level.

Although quitting smoking has been proven to be associated with lowering depression, anxiety, and stress, and improve positive mood and quality of life [27], less than 10% smokers successfully quit in the US [14]. Previous studies show that adult smokers who have a quit attempt are associated with worse health, such as psychological distress [28,29]. However, these people have a lower successful cessation rate [14] which may be related to the highly addictive properties of nicotine [28]. To our knowledge, few studies have focused on smokers who have made a quit attempt. Furthermore, little is known about the association between HRQOL and smoking status for different races with different income levels. Therefore, this study aimed to assess HRQOL among Louisianan public hospital patients across race, income level, and smoking status.

## 2. Methods

### 2.1. Study Design and Participants

This study applied a cross-sectional design using data from the 2017 Louisiana Tobacco Control Initiative (LA-TCI) patient survey. In 2017, whites represented 63.8% of Louisiana’s population, African Americans represented 32.6%, and others (American Indians/Alaska Natives, Asians, Hispanics/Latinos, and Native Hawaiians and other Pacific Islanders,) represented 3.6%. Also in 2017, the median income in Louisiana was US $46,710, the poverty rate (19.7%) was second highest in the nation, and only 23.4% of the population had a bachelor’s degree or higher [30]. The LA-TCI, housed within the Behavioral and Community Health Sciences Department of LSU Health Sciences Center–New Orleans School of Public Health, promotes a multi-level, translational, and transdisciplinary approach to cessation services and research. Grounded by the U.S. Public Health Service Clinical Practice Guideline for Treating Tobacco Use and Dependence, the LA-TCI employs the aforementioned approach at the recommended system, clinical, and patient levels of healthcare. The vision of the LA-TCI is to position itself as a national leader in the creation and application of knowledge through health systems services and research benefiting Louisianans. Its mission is to provide care for the most medically vulnerable residents, including low-income, under-and un-insured, less educated, and minority populations. The goal of the LA-TCI is to reduce the burden of tobacco use in state health care systems. The 2017 patient survey was conducted from January to February 2017 in eight public hospitals located in population centers across Louisiana. The paper-based, anonymous survey, collected through patient self-reporting HRQOL, tobacco use, provider treatment, quit attempts, and demographic information. Information from 1267 patients was collected. In this study, patients who were not black or African American, or white, or those who did not provide a response for race (*n* = 101) were excluded. Because patients who are Hispanic or Latino, Asian, American Indian/Alaska Native, and Native Hawaiian/Other Pacific Islander only represented 3.2% of patient survey participants, they were excluded from the study. Also excluded were patients who did not provide a response in regards to quit attempts (*n* = 58). The analyzed sample included 1108 participants. Our study protocol has been reviewed and approved by the Institutional Review Board of LSUHSC-NO (approval #7901).

### 2.2. Measurement of HRQOL

The EQ-5D classification system consists of five dimensions: mobility, self-care, pain/discomfort, anxiety/depression, and usual activities, with five levels of responses for each item (I have no problems “1”, I have slight problems “2”, I have moderate problems “3”, I have severe problems “4”, and I have extreme/severe or I am unable“5”) [21]. The EQ-5D health state or profile is created from the responses to the five EQ-5D dimensions, which can be converted into an index value [31]. To translate responses into a EQ-5D index value, we used US population-based preference weights (developed by the Agency for Healthcare Research and Quality [32]) to convert responses from the five dimensions (No, slight, moderate, severe and extreme) into three dimensions (No “1”, slight (slight and moderate) “2”, and severe (severe and extreme) “3”) [19]. Previous studies have established that estimated values are similar between the 5-level version of the EQ-5D and 3-level version [20,33]. With [3] levels for each dimension, 243 theoretically possible health states in the response were defined, with 11,111 being the best health state (perfect health), and 33,333 being the worst health state. A scoring algorithm calculated the EQ-5D index scores based on the US population-based preference weights [19]. These scores usually range from 0 to 1, with higher scores representing a better quality of life. However, the index score can be negative, which represents that these respondents have a worse quality of life than they have in real life [34]. The construct validity, reliability, and value set of the EQ-5D index scores have been documented in the US [19,20,32] UK, Germany, France, Denmark, Japan, The Netherlands, Spain, Thailand, and Zimbabwe [18].

### 2.3. Definitions of Smoking Status

Subjects were classified into four groups by smoking status: Never smoker, former smoker, current smoker with a quit attempt, and current smoker without a quit attempt. Never smoker consisted of respondents who reported that they smoked fewer than 100 cigarettes in their lifetime. Former smoker consisted of respondents who reported smoking ≥100 cigarettes in their lifetime, and currently, do not smoke cigarettes. Current smoker with a quit attempt consisted of respondents who reported smoking ≥100 cigarettes in their lifetime, presently smoking every day or some days, and stopped smoking for one day or longer due to trying to quit smoking in the past 12 months. Current smoker without a quit attempt consisted of respondents who reported smoking ≥100cigarettes in their entire lifetime and were currently smoking every day or some days [35].

### 2.4. Subject Demographics

Four demographic variables were considered: gender, age, income, and education. Gender was classified as female or male. Age was classified into three categories (18–44, 45–64, and higher than or equal to 65). Income level was organized into two categories (less than $20,000 and higher than or equal to $20,000). Education level was classified into two groups (high school or below (≤12 years) and college or beyond (>12 years)). Income and education level were classified into these two levels because most participants who utilize Louisianan public hospitals are of lower income and education levels.

### 2.5. Statistical Analysis

Descriptive statistical analyses were used to determine the distributions of respondents’ characteristics. Chi-square tests were used to test whether there were associations between the characteristics of respondents and race. For the EQ-5D score, regression diagnostics showed that linear regression was appropriate. Multivariable linear regression analyses were applied to compare the EQ-5D scores between current smokers and others while adjusting for age, sex, race, income, and education. Furthermore, based on previous literature, we conducted an analysis by stratifying patients by race and income. We also used a variance inflation factor (VIF) to detect the presence of multi-collinearity; a VIF value >10 indicated severe multi-collinearity in the regression model. In our models, there was no multi-collinearity; all VIFs were <5. We interpreted our model by the coefficients of smoking status in estimating the EQ-5D score. All analyses were performed using SAS version 9.4 (SAS Institute, Cary, NC, USA). All tests were two-sided, and the statistical significance level was set at 0.05 for all tests. 

## 3. Results

Table 1 summarizes the subject demographics of the study sample (*n* = 1,108). In the overall sample, 660 (60.6%) subjects were ages 45–64 years old; 726 (65.6%) were female; 710 (71.2%) had incomes <$20,000, and 787 (72.3%) had education levels of high school or below (≤12 years). Regarding smoking status, 453 (40.9%) of the patients were current smokers (no recent quit attempt), 240 (21.7%) had never smoked, 170 (16.2%) were former smokers, and 236 (21.3%) were current smokers with a quit attempt. Furthermore, 653 (58.9%) were black or African American, and 455 (41.1%) were white. Relative to whites, black or African Americans were more likely to be female (67.1%), lower-income (<$20,000) (74.5%), current smokers (48.4%) and have education levels of high school or below (≤12 years) (74.3%).

Table 2 presents the means of the EQ-5D scores for each variable and the results from the linear regressions evaluating explanatory variables for the EQ-5D scores. Black or African Americans (median score = 0.80) were associated with a higher EQ-5D score than whites (median score = 0.76) (adjusted coefficient=0.06, 95% CI: 0.03–0.10). In addition, patients with incomes ≥$20,000 (median score = 0.82) had a higher EQ-5D score than those with incomes <$20,000 (median score = 0.78) (adjusted coefficient = 0.08, 95% CI: 0.04–0.12). Compared to current smokers without a quit attempt (median score = 0.78), current smokers with a quit attempt (median score = 0.71) had a lower score (adjusted coefficient = −0.06, 95% CI: −0.12–−0.01).

Table 3 shows the comparison of EQ-5D scores across smoking status after stratification by race and income. Among black or African Americans with incomes <$20,000, current smokers with a quit attempt had lower EQ-5D scores than current smokers (adjusted coefficient = −0.12, 95% CI: −0.21– −0.04). Moreover, black or African Americans who never smoked and with incomes ≥ $20,000 had a tendency to have a higher EQ-5D score than those who were current smokers (adjusted coefficient *t* = 0.11, 95% CI: −0.01–0.22). However, there was no statistical significance across different smoking status for whites whose incomes were either <$20,000 or ≥$20,000.

## 4. Discussion

This study examined the associations between HRQOL and race, income level, and smoking status public hospital patients in Louisiana. Racial and income disparities for HRQOL between black or African Americans and whites were evident among adult patients from eight public hospitals. Black or African Americans had better HRQOL than whites, and lower-income patients had a worse HRQOL than their higher-income counterparts. In addition, current smokers with a quit attempt had a lower HRQOL than those without a quit attempt. After stratifying by race and income, black or African Americans who had lower-incomes and currently smoked with a quit attempt reported a more negative HRQOL than current smokers without a quit attempt.

Our results showed that both black or African Americans and whites had lower HRQOL than those in a previous study that focused on US adults with one or more chronic diseases [36]. A possible reason for this may be that our survey was conducted in primary care clinics in Louisiana public hospitals. The primary goal of public hospitals is to serve vulnerable populations who are on Medicaid (an insurance program to help low-income people of all ages [37]), Medicare (an insurance program to serve the elderly [37]), or who are uninsured. These minorities and lower-income populations are more likely to report worse HRQOL [38,39]. Also, patients included in our study were older and could have been less healthy than those in previous studies. A study by Craig and Rand used an online survey to collect data from 8222 US adults from all 50 States to estimate EQ-5D scores. In this investigation, respondents with poor health reported a low score (0.672) [20]. This may be another reason why there was a lower HRQOL in our study. We also observed that black or African Americans had higher HRQOL than whites. Previous studies have found that African Americans have a better psychological HRQOL due to higher levels of spirituality and social support [40,41,42]. In our study, 33.3% of black or African Americans reported that they had an anxiety or depression problem, and 48.6% of whites reported that they had such issues (unpublished data). Therefore, previous studies support our findings and provide a possible reason why black or African Americans reported better HRQOL than whites.

Our findings demonstrate that former smokers had a similar EQ-5D index to that of current smokers. A possible explanation for this could be that our study could not distinguish the level of smoking behavior among current smokers. Mulder and colleagues examined the effect the amount of smoking and the time between stopping smoking had on quality of life and found a difference in the quality of life between current heavy smokers and ex-smokers [43]. There was no difference between light and moderate smokers compared to ex-smokers [43]. Additionally, current smokers with a quit attempt had a worse HRQOL than current smokers without a quit attempt. This result is consistent with a previous study by McClave et al., which demonstrated a lower HRQOL among smokers who were unsuccessful in quitting [35]. McClave et al. used the Behavioral Risk Factor Surveillance System (BRFSS) to investigate the HRQOL across current smokers who unsuccessfully attempted to quit, current smokers who made no attempt to quit, former smokers, and individuals who never smoked. Their main findings show that current smokers who unsuccessfully attempted to quit were more likely to report mental distress, physical distress, and pain than those who made no attempts to stop [35].

In our study, black or African Americans who were on a lower-income, and current smokers with a quit attempt, had a lower HRQOL than those who were current smokers without a quit attempt. There are several possible explanations for the relationships between smoking status and HRQOL among lower-income black or African Americans. First, a low income is known to be associated with a lower HRQOL among African Americans [40]. Additionally, unsuccessful quitters tend to report decreased HRQOL relative to those who were non-quitters [35]. Therefore, because of this disparity, black or African Americans who are on a lower-income, currently smoke, but have tried to quit, should be identified as a discrete population. Third, these patients may have worse health, such as psychological distress, which may motivate them to attempt to quit smoking [28]. Smoking cessation interventions should target this population and provide effective cessation services to help them quit smoking and improve their HRQOL. Longitudinal research is necessary to understand the association between smoking status and HRQOL among specific populations.

There are several strengths to this study. First, our survey was conducted in a primary care setting. Thus, information from participants represents data collected in comparable healthcare delivery systems across the state, and everyone who responded to the survey had seen a healthcare provider across the three-month time frame of data collection. Second, this study used EQ-5D scores to represent HRQOL. EQ-5D scores have been validated [19] as appropriate for comparison when the scores are adjusted by population-based weights [33]. Third, this study considered race, income level, and smoking status to establish that lower-income black or African Americans with a quit attempt had a worse HRQOL. These findings provide more information than previous studies focusing only on the association between HRQOL and smoking status [35].

However, our study uses patient survey data, which may pose some limitations in regards to generalization. First, identifying or establishing a causal relationship between smoking status and HRQOL is not possible due to the cross-sectional nature of the survey data. Although we cannot infer that smoking leads to a poor HRQOL, [35] our findings show that current smokers with a quit attempt have a worse HRQOL than those without a quit attempt. A stronger association was also evident among lower-income black or African Americans, which supports the notion that a poor HRQOL may motivate people to attempt to quit smoking. Future studies are encouraged to conduct longitudinal research to understand the causality of this association. Second, self-reported data from patient surveys may be biased and tend to give narrow estimated associations. Third, respondents included only those from public hospitals, which might limit the generalizability of the results. Finally, our results could be confounded by unmeasured factors, such as chronic disease conditions, which have been associated with increasing attempts to quit among smokers [44] and with decreasing HRQOL [17,20].

## 5. Conclusions

There exists racial and income disparities in HRQOL. In our study, lower-income black or African Americans who were current smokers with a quit attempt reported poorer HRQOL. Smoking cessation interventions should focus on lower-income black or African American smokers with a prior quit attempt and provide tailored cessation services to address specific dimensions of their HRQOL. Additionally, future research focusing on interventions for smokers who have attempted to quit should explore factors associated with poor HRQOL. Strategies to reduce disparities in these populations must be developed. Future research could also consider evaluating the cost saved on care when boosting HRQOL via a cost-effectiveness analysis among lower-income black or African American smokers who had a quit attempt.

## Figures and Tables

**Table 1:** Descriptive statistics for whites, black or African Americans (*n* = 1108).

Variables	Overall (*n* = 1108)		Black or African American (*n* = 653)		White (*n* = 455)		*P*
	*n*	%	*n*	%	*n*	%	
Age							0.204
18-44	242	22.22	154	24.10	88	19.56	
45-64	660	60.61	377	59.00	283	62.89	
≥65	187	17.17	108	16.90	79	17.56	
Sex							0.213
Female	726	65.64	437	67.13	289	63.52	
Male	380	34.36	214	32.87	166	36.48	
Income							0.006
<US$20,000	710	71.21	438	74.49	272	66.50	
≥US$20,000	287	28.79	150	25.51	137	33.50	
Education							0.082
High school or below (≤12 years)	787	72.33	477	74.30	310	69.51	
College or beyond (>12 years)	301	27.67	165	25.70	136	30.49	
Smoker type							<0.001
Current smokers without a quit attempt	453	40.88	316	48.39	137	30.11	
Never smokers	240	21.66	116	17.76	124	27.25	
Former smokers	179	16.16	100	15.31	79	17.36	
Current smokers with a quit attempt *	236	21.30	121	18.53	115	25.27	

* Current smokers with a quit attempt were defined as respondents who reported smoking ≥100 cigarettes in their entire lifetime and reported now smoking every day or some days but stopped smoking for one day or longer in the past 12 months.

**Table 2:** Median of EQ-5D scores in each variable and multiple linear regression analysis.

Variables		Median	Q1	Q3	Crude Model					Adjusted Model		
					Coefficient	95% CI		*P*-Value		Coefficient	95% CI	*P*-Value
Race												
	White	0.76	0.46	0.83	0				0			
	Black or African American	0.80	0.46	1.00	0.06	0.02	0.09	<0.01	0.06	0.03	0.10	<0.01
Age												
	18-44	0.81	0.55	1.00	0				0			
	45-64	0.80	0.46	0.85	−0.05	−0.09	−0.01	0.01	−0.04	−0.08	0.01	0.08
	≥65	0.78	0.46	0.84	−0.02	−0.08	0.03	0.36	−0.02	−0.07	0.04	0.57
Sex												
	Female	0.78	0.40	0.84	0				0			
	Male	0.80	0.46	0.86	0.01	−0.03	0.04	0.99	0.01	−0.02	0.05	0.48
	Income											
	<US$20,000	0.78	0.46	0.84	0				0			
	≥US$20,000	0.82	0.69	1.00	0.08	0.04	0.12	<0.01	0.08	0.04	0.12	<0.01
	Education											
	High school or below (≤12 years)	0.78	0.46	0.84	0				0			
	College or beyond (>12 years)	0.78	0.47	0.93	0.02	−0.02	0.06	0.32	0.01	−0.03	0.05	0.57
	Smoker type											
	Current smokers without a quit attempt	0.78	0.46	0.84	0				0			
	Never smokers	0.81	0.51	1.00	0.04	−0.01	0.08	0.10	0.03	−0.02	0.08	0.21
	Former smokers	0.77	0.46	0.84	−0.01	−0.06	0.04	0.80	−0.01	−0.06	0.04	0.69
	Current smokers with a quit attempt ^*^	0.71	0.44	0.82	−0.07	−0.13	−0.02	0.01	−0.06	−0.12	−0.01	0.03

EQ-5D scores were calculated using the US population-based preference weights. These scores usually range from 0 to 1. Higher scores represent a better quality of life. However, it is possible that the index score will be negative, which represents people who have a worse quality of life than they have in real life. * Current smokers with a quit attempt were defined as respondents who reported smoking ≥100 cigarettes in their entire lifetime and reported now smoking every day or some days but stopped smoking for one day or longer in the past 12 months. Q1: First Quartile; Q3: Third Quartile.

**Table 3:** Stratifying race and income to compare EQ-5D scores across smoking status in linear regression models.

Variables	Coefficient	95% CI	*P*-Value
White			
<US$20,000			
Current smokers without a quit attempt (Reference)	0		
Never smokers	−0.02	−0.11–0.07	0.65
Former smokers	−0.02	−0.12–0.08	0.71
Current smokers with a quit attempt *	−0.10	−0.20–0.01	0.07
≥US$20,000			
Current smokers without a quit attempt (Reference)	0		
Never smokers	0.10	−0.02–0.23	0.11
Former smokers	0.04	−0.09–0.17	0.59
Current smokers with a quit attempt *	0.08	−0.06–0.23	0.27
Black or African American			
<US$20,000			
Current smokers without a quit attempt (Reference)	0		
Never smokers	0.01	−0.06–0.09	0.74
Former smokers	−0.05	−0.14–0.04	0.25
Current smokers with a quit attempt *	−0.12	−0.21–−0.04	<0.01
≥US$20,000			
Current smokers without a quit attempt (Reference)	0		
Never smokers	0.11	−0.01–0.22	0.06
Former smokers	0.03	−0.09–0.16	0.60
Current smokers with a quit attempt *	0.14	−0.04–0.33	0.13

All models were adjusted by age, gender, and education. EQ-5D scores were calculated using the US population-based preference weights. These scores usually range from 0 to 1. Higher scores represent a better quality of life. However, it is possible that the index score is negative, which represents people who have a worse quality of life than they have in real life. * Current smokers with a quit attempt were defined as respondents who reported smoking ≥100 cigarettes in their entire lifetime and reported now smoking every day or some days but stopped smoking for one day or longer in the past 12 months.
